# Sucrose and malic acid in the tobacco plant induce *hrp* regulon in a phytopathogen *Ralstonia pseudosolanacearum*

**DOI:** 10.1128/jb.00273-24

**Published:** 2025-02-04

**Authors:** Yuzhu Cao, Masayuki Tsuzuki, Akinori Kiba, Yasufumi Hikichi, Yong Zhang, Kouhei Ohnishi

**Affiliations:** 1 The United Graduate School of Agricultural Sciences, Ehime University, Ehime, Japan; 2 Faculty of Agriculture and Marine Science, Kochi University 12888 , Kochi, Japan; 3 College of Resources and Environment, Southwest University 597565 , Chongqing, China; Philipps-Universitat Marburg Fachbereich Biologie, Marburg, Germany

**Keywords:** *hrp* regulon, malic acid, sucrose, tobacco, RSSC

## Abstract

Genes encoding a type III secretion system in *Ralstonia pseudosolanacearum* are regulated by HrpB as an *hrp* regulon and induced only in plants. This study aimed to identify the plant signals that induce the *hrp* regulon and confirm the signal recognition mechanism. Signaling molecules that induce *hrpB* expression were screened using resting cells of the *hrpB-lacZ* reporter strain. Only the soluble fraction of smashed tobacco seedlings induced *hrpB* expression. The heated soluble fraction retained its *hrpB*-inducing activity, indicating that the signaling molecules were not proteins. When the soluble fraction was fractionated into acidic, neutral, and basic components, both the acidic and neutral fractions induced *hrpB* expression. As neutral compounds, sucrose, glucose, and fructose have been found to induce *hrpB* expression. Sucrose-induced *hrpB* expression was greatly reduced in the *prhA* mutant, indicating that the TonB-dependent receptor PrhA perceives sugars. Among the organic acids found in the acidic fractions, malic acid most efficiently induced *hrpB* expression, which was reduced by the mutation of a hybrid histidine kinase gene of a two-component system, *rsc1598*, indicating that Rsc1598 may sense malic acid. We demonstrated direct binding of Rsc1598 to malic acid using isothermal titration calorimetry.

**IMPORTANCE:**

Similar to other Gram-negative plant pathogens, the type III secretion system (T3SS) is the most important virulence factor in *Ralstonia pseudosolanacearum*. The genes for the T3SS are regulated as an *hrp* regulon, activated only when the pathogen encounters the plants, indicating that the pathogen must sense plant signals. For the first time, we identified two signaling compounds, sucrose and malic acid, that are abundantly found in tobacco roots. The *hrp* operon was induced even in non-host plants, possibly because sucrose and malic acid are common in plants. We also found that *R. pseudosolanacearum* membrane proteins received sucrose and malic acid independently. As a next step, antagonists of signaling molecules can be screened.

## INTRODUCTION

*Ralstonia solanacearum* species complex (RSSC) is a soil-borne bacterium that can cause disastrous bacterial wilt in hundreds of plant species, including important crops such as tobacco, tomato, potato, banana, eggplant, and pepper (1). RSSC is classified into three distinct species: *R. pseudosolanacearum* (phylotypes I and III), *R. solanacearum* (phylotypes IIA and IIB), and *R. syzygii* (phylotype IV) (2). Each phylotype corresponds to a geographic origin: phylotype I (Asia), phylotype II (Americas), phylotype III (Africa), and phylotype IV (Indonesia and Japan). Bacteria in soil and water environments move to plant hosts by sensing root exudates via chemotaxis (3, 4) and infecting host plants through wounds and natural openings of plant roots (5). As a soil pathogen, *R. pseudosolanacearum* initially invades the root system of the host plant and colonizes the roots during the early stages.

When RSSC invades the host plant, the gene expression pattern changes significantly compared with saprophytic growth in the soil (6, 7). Similar to several Gram-negative bacteria, the syringe-like type III secretion system (T3SS) is the main pathogenic system in RSSC. RSSC produce a range of virulence factors, including exopolysaccharide (EPS), cell wall-degrading enzymes, and dozens of type III effectors (T3Es), which are injected into host cells via T3SS (8). Mutant strains that cannot construct T3SS and secrete T3Es completely lose pathogenicity (9).

Genes encoding T3SS components are clustered and regulated as an *hrp* regulon in RSSC phylotype-I *R. pseudosolanacearum* strains (10). The expression of the entire *hrp* regulon is controlled by HrpB, an AraC family transcriptional regulator, and is activated only *in planta* or in *in vitro* nutrient-poor conditions mimicking the plant apoplast (11), suggesting that plant signals must be sensed by bacteria for *hrp* regulon induction.

Several receptor proteins are involved in Gram-negative bacteria. These include TonB-dependent receptors (TBDRs) in the outer membrane and the sensor histidine kinase of the two-component system in the inner membrane. TBDR is mainly associated with the absorption of iron and vitamin B_12_ (12). A signaling cascade initiating from PrhA to the response regulator HrpG, PrhA-PrhR/I-PrhJ-HrpG has been proposed (Fig. S1) (13, 14). Although PrhA belongs to the TBDR family, iron is not sensed by PrhA (15). PrhI is an ECF sigma factor, and the membrane protein PrhR is an anti-sigma factor. In the absence of an exogenous signal(s), PrhI binds to PrhR. Once the signal(s) are received by an outer membrane protein, PrhA, the information is transferred to PrhR. Consequently, PrhI is released into the periplasm where it activates *prhJ* transcription as a sigma factor. PrhJ is a transcriptional regulator that activates *hrpG* expression. Under *in vitro* nutrient-poor conditions mimicking the plant apoplast, unknown small nutrient signals can activate HrpG activity independent of the PrhA signal cascade (16).

The two-component system pairs the inner membrane protein histidine kinase and response regulator. Histidine kinase is autophosphorylated after sensing external signal(s) and phosphorylates the response regulator to control the cellular response. The expression of *hrpB* is positively regulated by two paralogous response regulators, HrpG and PrhG, in parallel ways (17; Fig. S1). Although cognate sensor kinases have not yet been identified, phosphorylation of HrpG and PrhG in response to host signals is important for their functions (18, 19).

Although *R. pseudosolanacearum* proteins involved in *hrp* regulon expression have been studied in detail, no information regarding the plant signaling molecules that induce the *hrp* regulon is available. In this study, we attempted to identify plant signaling molecules in *Nicotiana benthamiana* seedlings using the *hrpB-lacZ* reporter strain.

## RESULTS

### Expression of the *hrpB* gene was specifically induced in response to soluble non-protein plant components

The resting cells of the *hrpB-lacZ* reporter strain were used to identify potential plant signaling molecules that induce *hrpB* gene expression. Once *hrpB* is induced, the transcriptional activator HrpB activates the *hrp* regulon. Resting cells were used as whole-cell biocatalysts. They retained their enzymatic activities without continuous growth. Resting cells were freshly prepared by growth in a nutrient-rich medium to the log phase, washed several times, and suspended in a buffer without a carbon source. We used resting cells instead of growing cells to monitor gene expression in the *hrpB-lacZ* reporter strain. Resting cells retain their enzymatic activity without continuous growth. The resting cells remained alive after 20 h of incubation at room temperature (Fig. S2). Freshly prepared resting cells were 1.4 × 10^8^ CFU/mL. After 20 h, the cell number slightly increased to 4.2 × 10^8^ CFU/mL.

**Fig 1:**
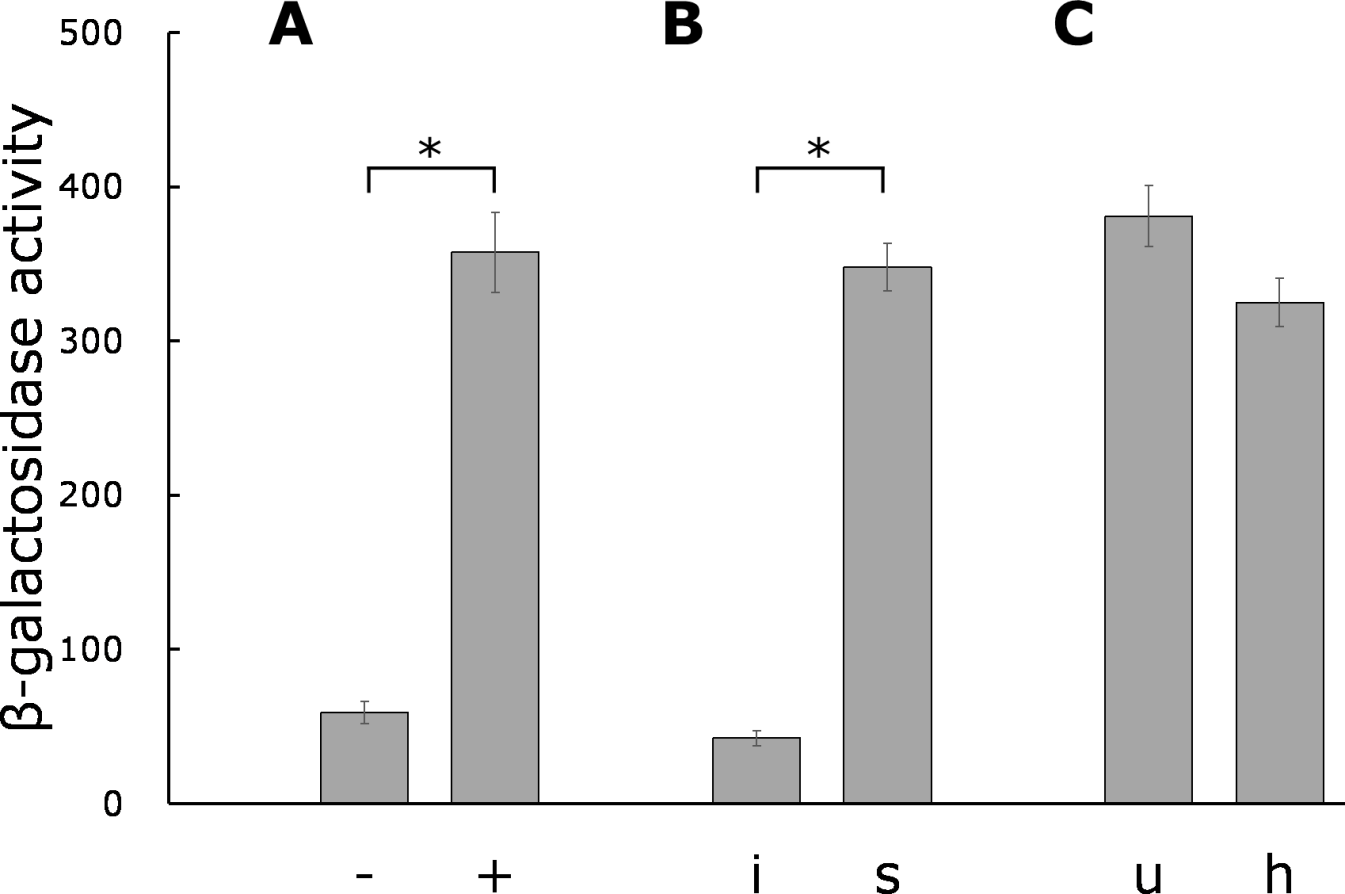
Induction of *hrpB* expression in tobacco extracts in a resting cell assay. Cells were grown to an OD_600_ of approximately 0.5 in rich B medium, washed twice, and resuspended in the 1/4M63 buffer (25 mM KH_2_PO_4_, 3.75 mM (NH_4_)_2_SO_4_, 0.25 mM MgSO_4_, 0.45 µM FeSO_4_) to the OD_600_ of 1.0. Cells (0.1 mL) were used in a 1-mL reaction mixture in a 96-well deep-well plate. Tobacco extract (0.1 mL) was added, and the reaction was continued at 25°C for 20 h. After the reaction, β-galactosidase activity was measured using the 0.2-mL mixture with ONPG as a substrate, as shown in the Miller unit. (**A**) Extraction buffer (−) and smashed tobacco extracts (+) were mixed with resting cells. (**B**) Smashed tobacco extracts were centrifuged to separate the insoluble and soluble fractions. The insoluble fraction was resuspended in the extraction buffer. The insoluble (**I**) and soluble (**S**) fractions were mixed with resting cells. (**C**) The soluble fraction was heated at 100°C for 30 min and centrifuged to remove denatured proteins. sSoluble fractions without heating (**U**) and with heating (**H**) were mixed with resting cells. Data are presented as mean ± SD of three independent experiments (unpaired *t*-test, *P < 0.05).

Aseptically grown seedlings of *N. benthamiana* were crushed by bead beating and mixed with *hrpB-lacZ* resting cells for 20 h at room temperature. β-Galactosidase activity was measured using a standard method with ONPG as a substrate. Optical density (OD_600_) values estimating cell numbers in the reaction mixture ranged from 0.1 to 0.2, and no significant OD_600_ differences were observed between the reaction mixtures with or without tobacco extracts. *hrpB* expression was significantly induced by the tobacco extract (Fig. 1A). When the tobacco extracts were separated into soluble and insoluble components, only the soluble components induced *hrpB* expression (Fig. 1B). Even after the soluble components were heated at 100°C for 30 min, they retained their induction activity (Fig. 1C), indicating that the potential plant signaling molecules might not be proteins.

### Potential plant signaling molecules were either acidic or neutral but not basic

**Fig 2:**
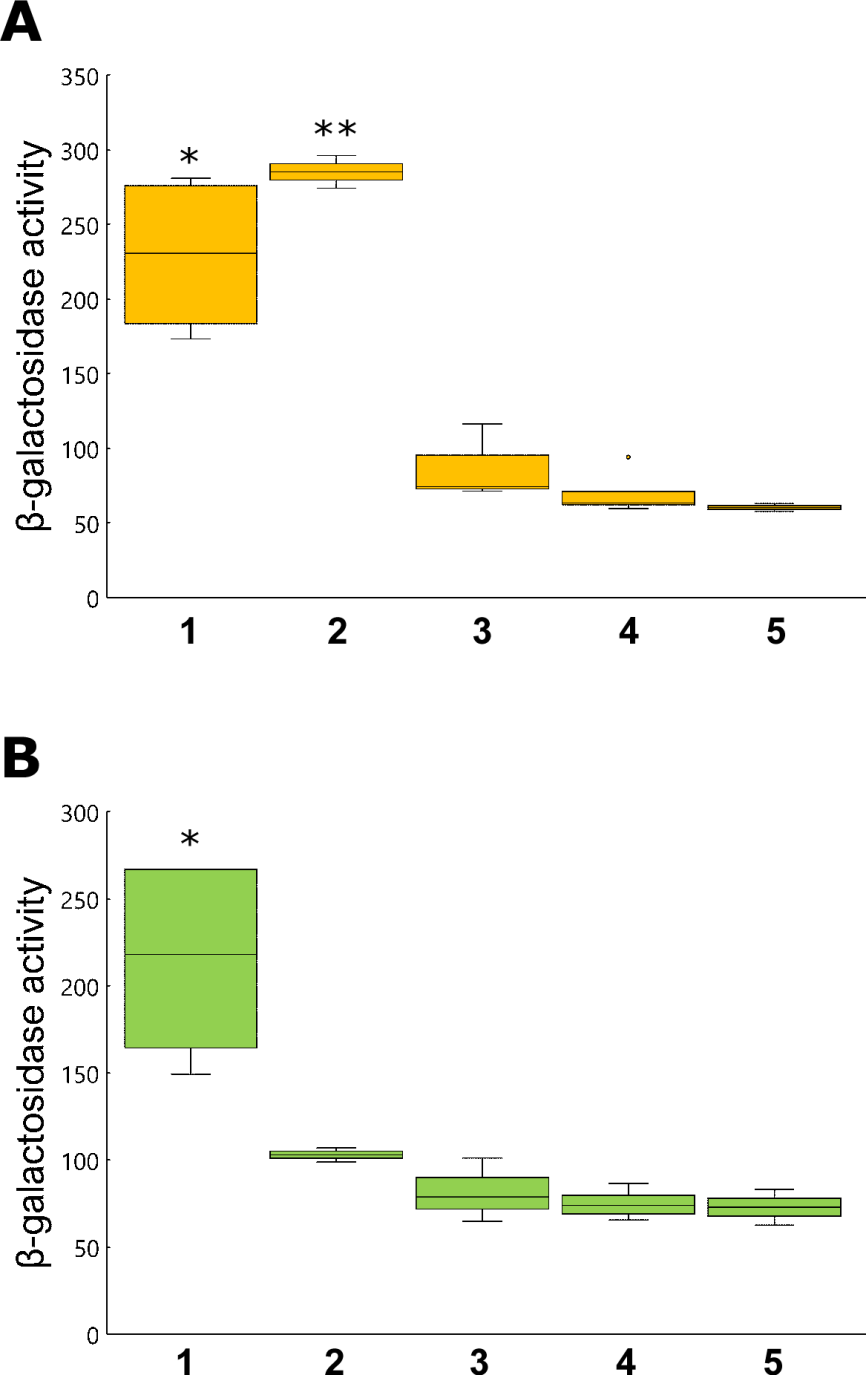
Separation of tobacco extracts using Sep-PAK C_18_. The soluble fractions of tobacco extracts derived from the roots (**A**) and leaves (**B**) of *N. benthamiana* were loaded onto Sep-PAK C_18_ cartridges. After washing with extraction buffer (25 mM KH_2_PO_4_, 3.75 mM (NH_4_)_2_SO_4_, 0.25 mM MgSO_4_, 0.45 µM FeSO_4_), bound compounds were eluted with methanol in a stepwise gradient. *hrpB* expression in resting cells was measured as described in Fig. 1, with samples of 1: flow through, 2: wash, 3: elution with 50% methanol, 4: elution with 100% methanol, and 5: none. Box plots show the medians (horizontal line in the box), 25% and 75% quartiles, max/min values, and outliers marked as circles. ** and * indicate a statistically significant difference from none at *P* < 0.01 and *P* < 0.05. The experiments were repeated at least three times.

**Fig 3:**
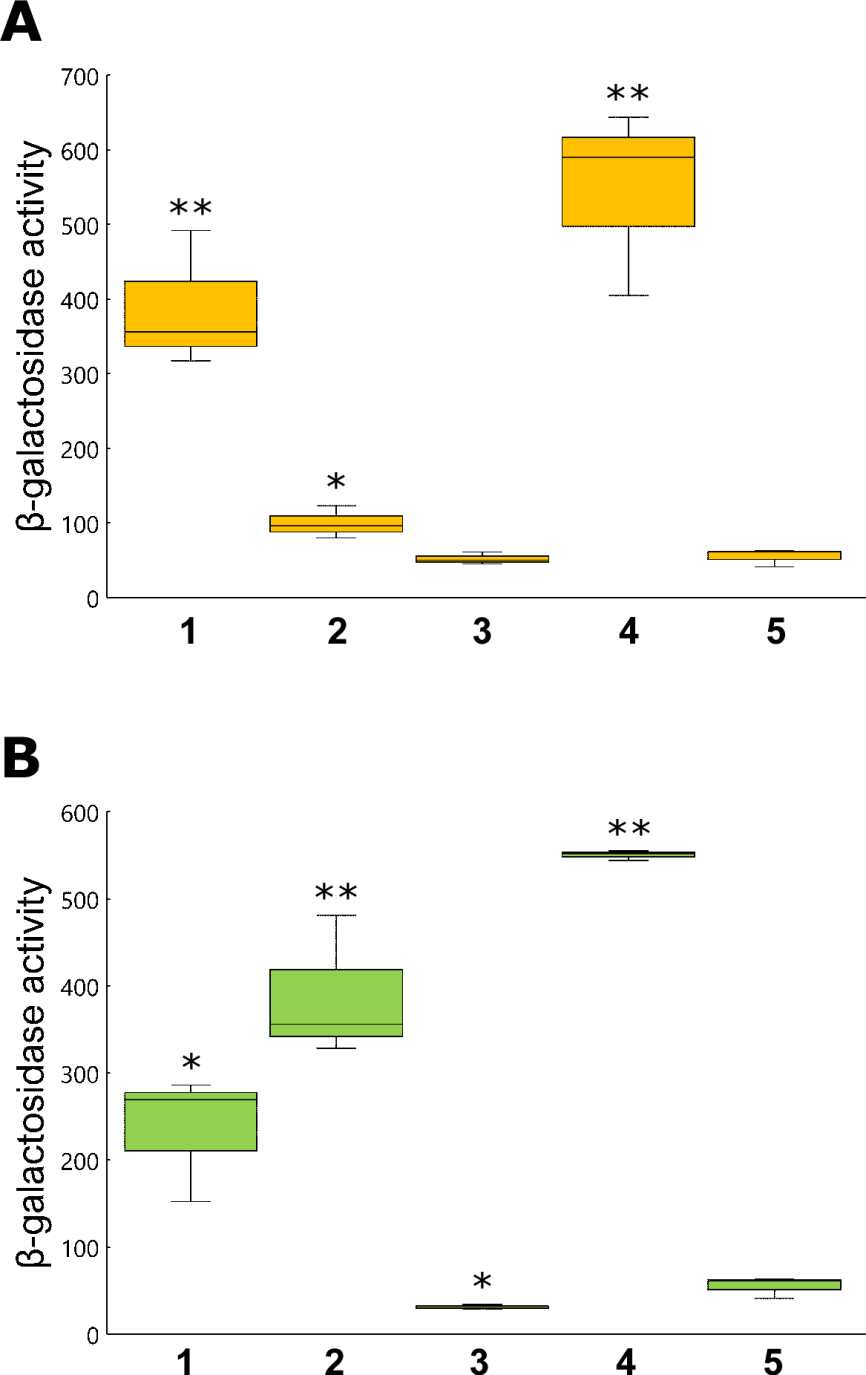
Separation of tobacco extracts using tandem Sep-PAK CM and QMA cartridges. Flow-through and wash fractions after a Sep-PAK C_18_ cartridge of tobacco extracts derived from roots (**A**) or leaves (**B**) were loaded on Sep-Pak CM and QMA cartridges connected in tandem. The two cartridges were disconnected after washing with extraction buffer, and the bound components were eluted separately with elution buffer (1 M NaCl, 10 mM ammonium acetate, pH 5.5 in 20% acetonitrile). *hrpB* expression in resting cells was measured as described in Fig. 1, with samples 1: flow through, 2: wash, 3: elution from the CM cartridge, 4: elution from the QMA cartridge, and 5: none. Box plots show the medians (horizontal line in the box), 25% and 75% quartiles, max/min values, and outliers marked as circles. ** and * indicate a statistically significant difference from none at *P* < 0.01 and *P* < 0.05. The experiments were repeated at least three times.

Soluble non-protein fractions obtained from tobacco roots or leaves were applied to a solid-phase extraction (SPE) Sep-Pak C_18_ cartridge (Fig. S3), which extracts nonpolar compounds from an aqueous sample (20). When the plant extracts were subjected to Sep-Pak C_18_, *hrpB* expression was greatly induced by the flow-through and wash fractions in the root extracts (Fig. 2A) and by the flow-through fraction in the leaf extracts (Fig. 2B). Since both flow-through and wash fractions were components that did not bind to Sep-Pak C_18_, the signaling molecules were considered polar compounds. The tandem cation/anion exchange SPE cartridge method was used for further separation (21). Polar compounds that did not bind to Sep-Pak C_18_ were applied to the Sep-Pak cation-exchange cartridge CM and anion-exchange cartridge QMA, and separated into basic, neutral, and acidic compounds (Fig. S3). The flow-through and wash fractions after tandem cartridges corresponded to neutral compounds. The CM- and QMA-bound fractions corresponded to basic and acidic compounds, respectively. *hrpB* expression was greatly induced by flow through, washing, and QMA-bound fractions in both leaf and root extracts (Fig. 3A and B). This suggests that potential plant signaling molecules are neutral and acidic.

### Several organic acids identified in tobacco tissues induced the *hrpB* expression

**Fig 4:**
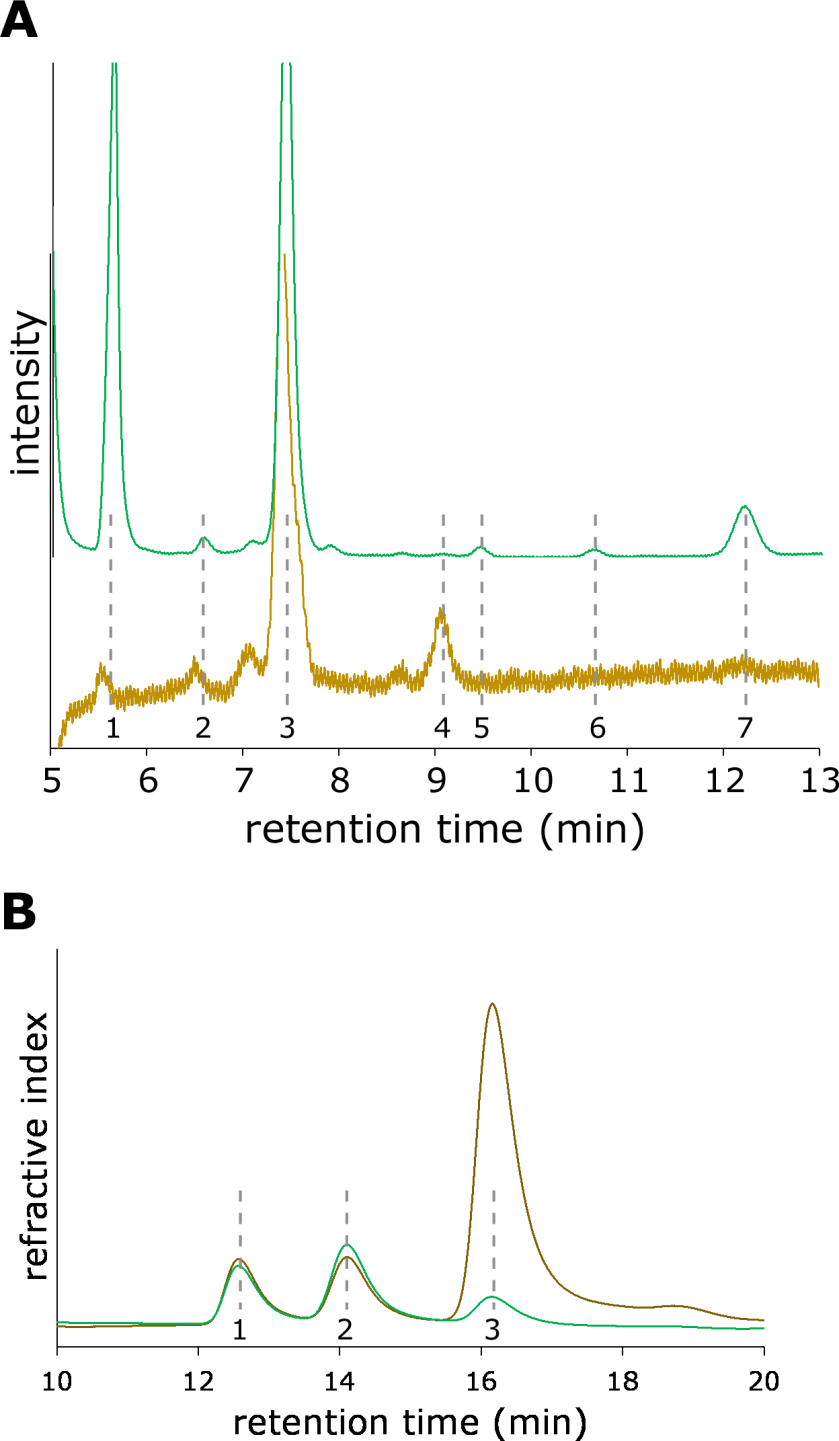
Identification of compounds in tobacco extracts. (**A**) Acidic compounds from roots (brown line) and leaves (green line) were separated using TSKgel SCX equilibrated with 3 mM HClO_4_ at a flow rate of 1 mL/min. Organic acids (1: oxalic acid, 2: citric acid, 3: malic acid, 4: succinic acid, 5: formic acid, 6: acetic acid, 7: L-pyroglutamic acid) are indicated. (**B**) Neutral compounds from the roots (brown line) and leaves (green line) were separated using a Sugar-D column equilibrated with 75% acetonitrile at a flow rate of 1 mL/min. Sugars (1, fructose; 2, glucose; and 3, sucrose) are indicated.

Organic acids produced by host plants have biostimulatory effects on soil pathogenic bacteria. We speculated that the acidic compounds that affect *hrpB* expression are organic acids. QMA-bound compounds were applied to a strong cation-exchange column, TSKgel SCX, using a post-column method to separate and detect organic acids (22). We mainly observed seven organic acids, oxalic, citric, malic, succinic, formic, acetic, and L-pyroglutamic acids, in the tobacco acidic extracts (Fig. 4A).

### Three sugars were mainly identified in tobacco extracts

We speculate that sugars are the neutral compounds that induce hrpB expression. Neutral compounds were applied to a sugar-D column (Nacalai Tesque) for sugar analysis to separate sugars (23). The sugar-D column had a polyamine-based stationary phase, which resulted in different selectivity from that of the aminopropyl-based stationary phase. We found three sugars, fructose, glucose, and sucrose, in the neutral compounds in the leaf and root extracts (Fig. 4B). In the roots, the sucrose content was higher than that of other sugar components.

### Induction of *hrpB* expression with organic acids

**Fig 5:**
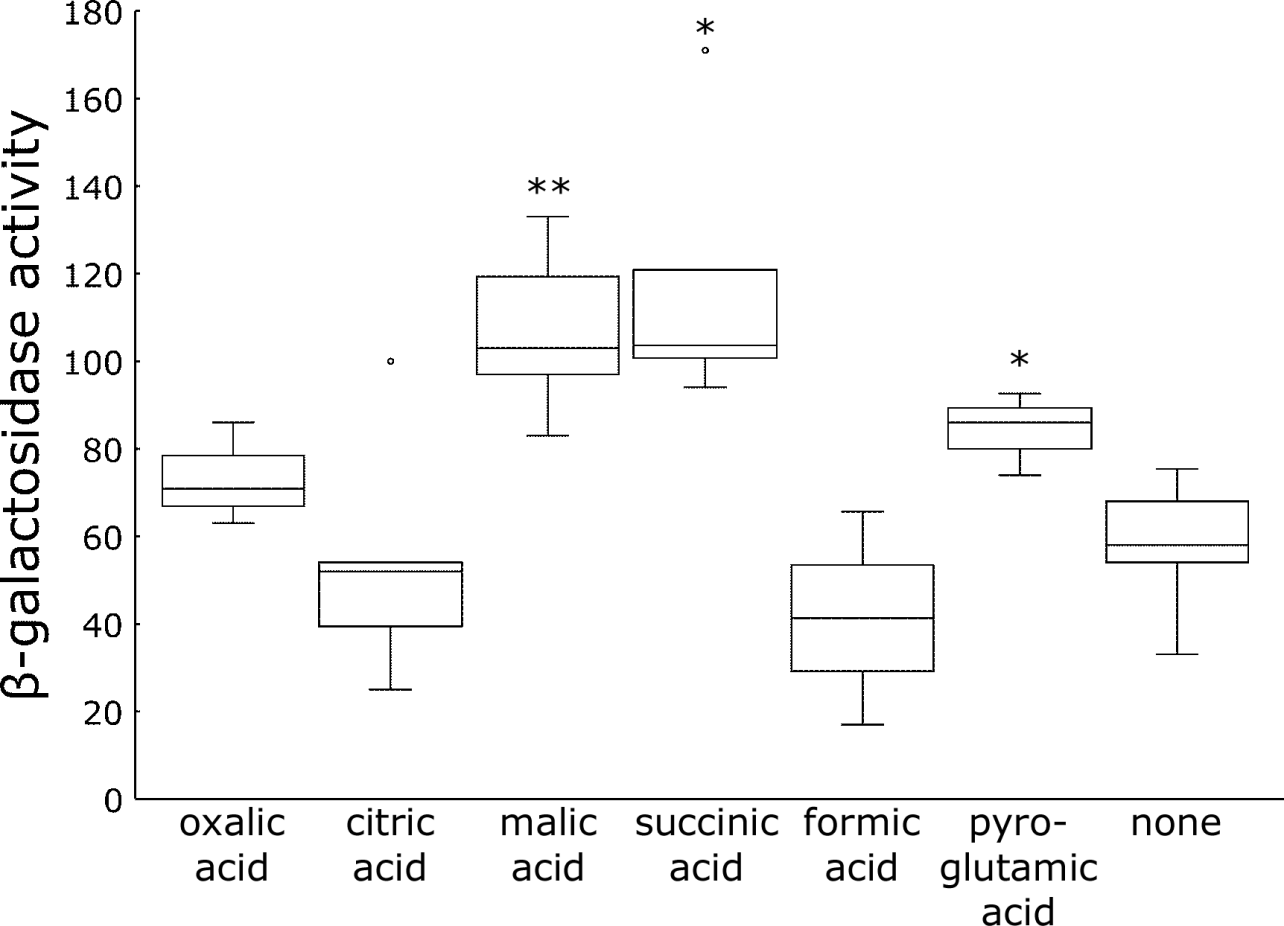
Induction of *hrpB* expression by organic acids. Individual organic acids were added to the reaction mixture at 0.1 mM. The total volume of the reaction mixture was 1 mL, containing 0.1 mL resting cells. The reaction continued at 25°C for 20 h. Box plots show the medians (horizontal line in the box), 25% and 75% quartiles, max/min values, and outliers marked as circles. ** and * indicate a statistically significant difference from none at *P* < 0.01 and *P* < 0.05. The experiments were repeated at least three times.

Among the organic acids found in tobacco extracts, individual pure organic acids, namely, oxalic, citric, malic, succinic, formic, and L-pyroglutamic acids, were added to *hrpB-lacZ* resting cells. Among these, malic, succinic, and L-pyroglutamic acids induced high levels of *hrpB* expression (Fig. 5). Among the six tested organic acids, malic acid was the most abundant in roots (Fig. 4A). We speculated that malic acid functions as a signaling molecule.

When freshly prepared resting cells (1.4 × 10^8^ CFU/mL) were mixed with malic acid or sucrose, the cell numbers increased to 6.8 × 10^8^ and 8.2 × 10^8^ CFU/mL, respectively (Fig. S2). These data indicated that malic acid or sucrose did not affect the viability of resting cells for at least 20 h of incubation. To further validate our resting cell assay, we monitored *hrpB-lacZ* expression in the growing cells. Overnight-grown RK5046 cells (*hrpB-lacZ*) were freshly diluted 100-fold in quarter-strength M63 medium with 0.25% glucose. After 5 h of incubation, the β-galactosidase activity was measured. Because glucose induced *hrpB* expression without adding any substances, *hrpB-lacZ* activity was relatively high (Fig. S4). When sucrose was added to the medium, minimal induction was observed. Malic acid addition induced *hrpB* expression as observed in resting cells. These results indicate that *hrpB* induction assay using resting cells is as effective as that using growing cells.

### PrhA might be involved in sugar recognition

**Fig 6:**
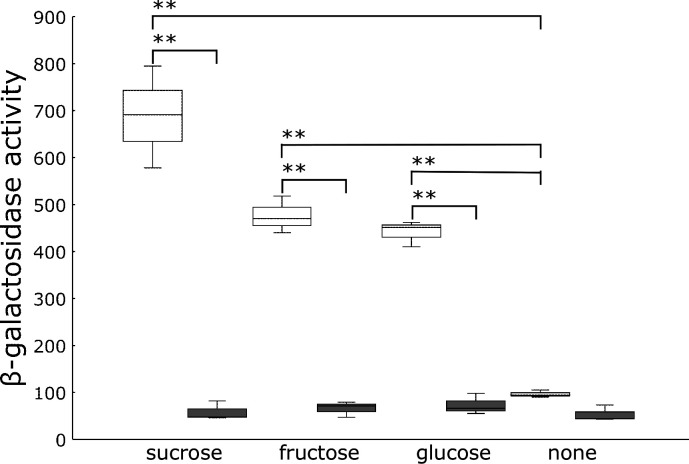
Induction of *hrpB* expression by sugars. Individual sugars were added to the reaction mixture at 0.01% with *hrpB-lacZ* (open box) or *hrpB-lacZ* Δ*prhA* (closed box). The reaction conditions are shown in Fig. 5. Box plots show the medians (horizontal line in the box), 25% and 75% quartiles, max/min values, and outliers marked as circles. **A statistically significant difference from none at *P* < 0.01. The experiments were repeated at least three times.

Fructose, glucose, and sucrose were found mainly in the tobacco extracts. Pure sugars, fructose, glucose, and sucrose were added to the *hrpB-lacZ* resting cells. Although all three sugars induced *hrpB* expression, sucrose was the most effective sugar (Fig. 6A). Because sucrose was abundant in the root extracts (Fig. 4B), it could be a signaling molecule that induces *hrpB* expression in the root, where *R. pseudosolanacearum* resides in the early infection stage.

PrhA initiates a signaling cascade in *R. pseudosolanacearum*. PrhA is an outer-membrane TBDRs that was first identified as an importer of the Fe^3+^ siderophore complex (24). TBDRs are responsible for importing non-Fe compounds including sugars (25). Therefore, we investigated the role of PrhA in sugar recognition in this study. The *prhA* gene was deleted in the *hrpB-lacZ* reporter strain and subjected to resting cell assay. Sugar-induced *hrpB* expression was completely lost in *prhA* mutants (Fig. 6). Among the three sugars, sucrose was the most abundant in roots (Fig. 4B). Taken together, TBDR PrhA may be involved in receiving sugars, mainly sucrose, as signaling molecules.

### Rsc1598, encoding a sensor kinase, was involved in the reception of organic acids

**Fig 7:**
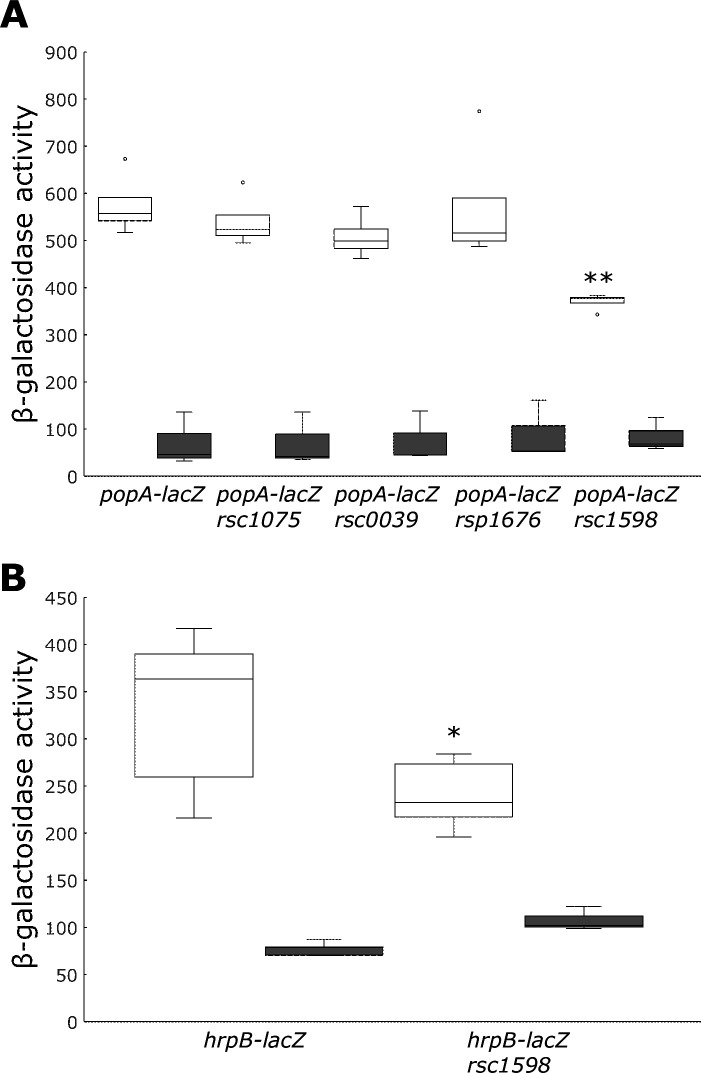
Involvement of the histidine kinase gene in *hrp* gene expression induced by malic acid. (**A**) The assay was conducted with 0.5 mM malic acid (open box) and none (closed box) using resting cells of *popA-lacZ*, *popA-lacZ* Δ*rsc1075*, *popA-lacZ* Δ*rsc0039*, *popA-lacZ* Δ*rsp1676*, and *popA-lacZ* Δ*rsc1598* strains. (**B**) The assay was conducted with 0.5 mM malic acid (open box) and none (closed box) using resting cells of *hrpB-lacZ* and *hrpB-lacZ* Δ*rsc1598* strains. Box plots show the medians (horizontal line in the box), 25% and 75% quartiles, max/min values, and outliers marked as circles. ** and * indicate a statistically significant difference from wild-type background at *P* < 0.01 and *P* < 0.05. The experiments were repeated at least three times.

The *popA* operon belongs to the *hrp* regulon and is regulated by HrpB (16). RK5050 is a *popA-lacZ* reporter strain of *R. pseudosolanacearum* OE1-1. We obtained a mutant library for *R. pseudosolanacearum* OE1-1 histidine kinase genes as a laboratory stock. The library consisted of 44 strains, each with a deletion of one of the histidine kinase genes in the RK5050 background. Among these, mutants of four histidine kinase genes, *rsc1075*, *rsc0039*, *rsp1676*, and *rsc1598*, showed slightly reduced *popA* expression when inoculated into *N. benthamiana* leaves (unpublished data). Therefore, we speculate that these histidine kinase genes may be involved in organic acid recognition. The expression of *popA* was monitored in resting cells of the four strains treated with malic acid (0.5 mM). The *rsc1598* mutation significantly reduced *popA* expression compared to the wild type (Fig. 7A). When the *rsc1598* mutation was introduced into the *hrpB-lacZ* reporter strain, resting cells of the mutant showed reduced *hrpB* expression (Fig. 7B), indicating that Rsc1598 might perceive malic acid.

### Malic acid bound to histidine kinase Rsc1598

**Fig 8:**
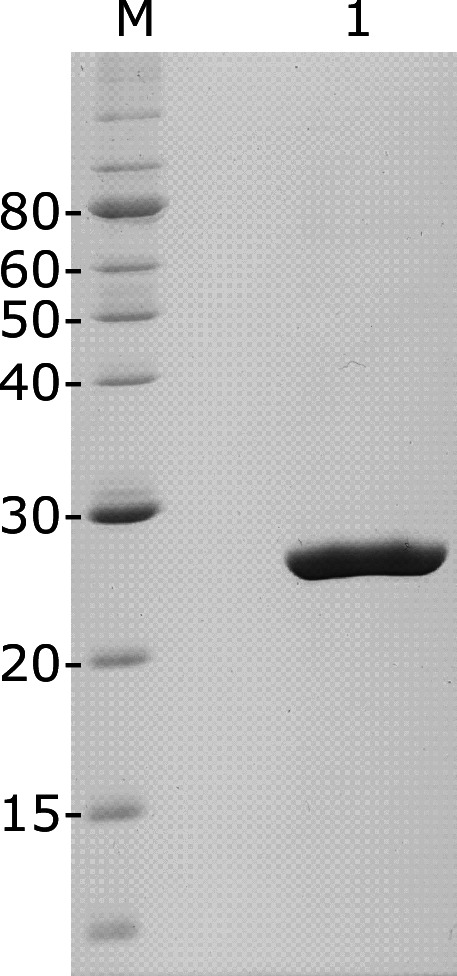
Purification of the periplasmic domain of histidine kinase RSc1598. *Escherichia coli* BL21(DE3) with plasmid ppet1598 was incubated at 37°C for 2 h, and then 0.1 mM IPTG was added. The cells were then incubated overnight at 28°C. The cleared lysate was prepared by sonicating the harvested cells and mixing them with Ni-NTA agarose gel. The bound protein was eluted with 250 mM imidazole. The purified protein was analyzed by 12.5% SDS-PAGE. Lane M, molecular mass marker; lane 1, purified RSc1598 (5 µg). The molecular masses are shown on the left (kDa).

Since Rsc1598 is a membrane protein histidine kinase, it is difficult to overexpress and purify it in *E. coli*. We attempted to express the periplasmic domain of Rsc1598, which might be a ligand-binding site (26). The periplasmic domain of Rsc1598 with a His-tag at the C-terminus was overexpressed in *E. coli* and purified to homogeneity using an Ni-NTA resin (Fig. 8).

We performed isothermal titration calorimetry (ITC) binding studies using the purified periplasmic domain of Rsc1598 and organic acids. ITC measures binding interactions by detecting the heat absorbed or released during binding (27). The integrated heat per injection (normalized to the concentration per mole of malate and corrected for the heat of dilution) was plotted against the molar ratio of malic acid to Rsc1598 (Fig. S5). Based on the relationship between the molar ratio and Δ*H* (Fig. S5), the *K*_*d*_ value for malic acid binding to the Rsc1598 periplasmic domain was 71.7 µM ( Table 1). The binding was driven by favorable enthalpy (Δ*H* = −1.61 kcal/mol) and entropy changes (*T*Δ*S* = −22.1 kcal/mol). The *K*_*d*_ values for succinic acid and L-pyroglutamic acid were 109.8 and 92.7 µM, respectively. These three organic acids, with lower *K*_*d*_ values, bound to the periplasmic domain of Rsc1598 with higher affinities than oxalic, citric, and formic acids. This is consistent with data showing that malic, succinic, and L-pyroglutamic acids induced *hrpB-lacZ* expression. These results demonstrate that the histidine kinase Rsc1598 may sense organic acids, especially malic acid, in the roots to induce the *hrp* regulon. Although fumaric acid, a member of the C4-dicarboxylic acid family along with malic and succinic acids, was not detected in tobacco plants by our HPLC system, the *K*_*d*_ value for fumaric acid was smaller than that of malic acid (Table 1).

**TABLE 1:** Thermodynamic data obtained by ITC for the binding of organic acids to the periplasmic domain of Rsc1598^*a*^

Ligand		*K*_*d*_ (μM)	−∆*H* (kcal mol^−1^)	−*T*∆*S* (kcal mol^−1^)	–∆*G* (kcal mol^−1^)
Malic acid	4 mM	68.4	1.9	21.9	23.8
	4 mM	64.3	1.47	22.5	24
	5 mM	89	1.44	21.7	23.1
	6 mM	65	1.61	22.3	23.9
Average		±11.7	±0.21	±0.37	±0.41
Succinic acid	5 mM	29.5	1.21	24.7	25.9
	6 mM	190	2.22	19	21.3
Average		109.8 ± 113.5	1.72 ± 0.71	±3.3	21.9 ± 4.0
L-pyroglutamic	4 mM	41	0.747	24.3	25.1
acid	5 mM	43.2	0.577	24.4	24.9
	6 mM	194	1.19	20	21.2
Average		92.7 ± 87.7	0.84 ± 0.32	22.9 ± 2.5	23.7 ± 2.2
Oxalic acid	4 mM	229	3.54	17.3	20.8
	6 mM	151	1.51	20.3	21.8
Average		190.0 ± 55.2	2.53 ± 1.44	18.8 ± 2.1	±0.7
Citric acid	4 mM	137	4.36	17.7	22.1
	5 mM	84.5	2.49	20.8	23.3
	6 mM	243	4.41	16.2	20.6
Average		154.8 ± 80.7	3.75 ± 1.09	±2.4	.0±1.4
Formic acid	5 mM	71.5	1.07	22.6	23.7
	6 mM	231	1.39	19.4	20.8
Average		±112.8	±0.23	.0±2.3	±2.1
Fumaric acid	4 mM	47	1.14	23.6	24.7
	6 mM	56.3	2.01	22.3	24.3
Average		±6.6	±0.62	.0±0.9	±0.3

^*a*^ The experiments were performed at 25°C in 50 mM sodium phosphate buffer (pH 7.6). The values for binding stoichiometry, *K*_*d*_, and ∆*H* were derived from a nonlinear least-squares fit according to a single-site binding model.

## DISCUSSION

RSSC are plant pathogenic bacteria with strong root colonization capabilities and positive chemotaxis toward host exudates (4). This study explored the plant signaling molecules sensed by *R. pseudosolanacearum* OE1-1 to induce *hrp* regulon. OE1-1 responded to several organic acids and sugars extracted from the tobacco leaves and roots. Malic acid and sucrose are the most significant plant-derived signaling molecules that induce *hrpB* expression. Plant-soluble extracts are divided into two categories: low-molecular-weight compounds, including various organic acids, sugars, amino acids, and secondary metabolites, and high-molecular-weight compounds, including proteins and polysaccharides (28, 29). To date, most host signal species identified in rhizosphere bacteria are low-molecular-weight compounds, and many studies have shown that soil bacteria can use these compounds in host plant cells to enhance pathogenicity. For example, RSSC use exogenous organic acids and amino acids from tobacco to enhance the expression of their chemotaxis and motility-related genes (30). Sucrose catabolism serves as a carbon source to provide energy and facilitate bacterial colonization of tomato stems (31).

Malic acid plays an important role in plant–microbial interactions. Malic acid secreted by the roots of *Arabidopsis thaliana* selectively signals and recruits the beneficial root bacterium *Bacillus subtilis* FB17 in a dose-dependent manner (32). *B. subtilis* RR4, which infects rice, also has a chemotactic effect on malic acid and can be manipulated to induce malic acid synthesis in rice roots (33). Furthermore, *Azospirillum brasilense* prefers malic acid and several other organic acids such as lactate, fumarate, and succinate as carbon sources (34). Malic acid is the second most preferred carbon source after glucose for organisms such as *B. subtilis* and *A. brasilense* (35, 36).

The leaf extracts contained more variation in organic acids than the root extracts did. Oxalic, citric, malic, formic, acetic, and L-pyroglutamic acids were detected in the leaf extracts. In contrast, oxalic, citric, malic, and succinic acids were detected in the root extracts. Malic acid and succinic acid were abundant in the roots. Organic acids and fatty acids regulate plant growth, act as chemical attractants, and stimulate microbial growth (37). Benzoic and 3-phenylpropanoic acids in tobacco root exudates stimulate the growth of *R. solanacearum* (38). Cinnamic, myristic, and fumaric acids promote *R. solanacearum* colonization and accelerate disease progression in tobacco (30).

Sucrose is the most common sugar in plant roots and shoots and is also an important carbon source in the first plant nutrient interface encountered by soil pathogens after invading the host (39). When RSSC colonize the interior spaces of plants and multiply, sucrose is the preferred carbon source (40). Bacteria also require sucrose to achieve full virulence in tomato xylem (6).

Rsc1598 contains a conserved protein domain family, COG4191, a signal transduction histidine kinase that regulates the C4-dicarboxylate transport system. Since we discovered that three members of C4-dicarboxylate, malic, succinic, and fumaric acids, interacted with Rsc1598, the binding of these organic acids to the sensor kinase Rsc1598 might activate the C4-dicarboxylate transport system along with *the hrp* regulon. There is a response regulator gene, *rsc1597*, next to *rsc1598*, and Rsc1597 might be a target of the histidine kinase Rsc1598 for phosphotransfer. We speculated that Rsc1598 might phosphorylate the response regulators HrpG or PrhG after receiving the plant signaling molecule, malic acid. We will perform phosphotransfer experiments from purified Rsc1598 to HrpG, PrhG, or Rsc1597 in the future.

In conclusion, we identified three types of organic acids (malic, succinic, and L-pyroglutamic acids) and three types of sugars (fructose, glucose, and sucrose) in tobacco extracts that induce the *hrp* regulon. Among these plant signaling molecules, malic acid and sucrose are considered the best inducers, and malic acid binds directly to histidine kinase Rsc1598. Once such plant signaling molecules are identified, antagonists can be screened for signaling molecules to control wilt disease. Antibiotics are used to control the growth of microorganisms. However, the emergence of drug-resistant strains remains problematic. The demand for chemicals that can control pathogens rather than antibiotics is increasing. One such example is the analog of an autoinducer, 3-OH MAME or 3-OH MAME, which is synthesized by *R. pseudosolanacearum* in a quorum sensing-dependent manner. Analog inhibitors of 3-OH MAME have been designed and are effective in controlling *R. pseudosolanacearum* infections (41). Analog inhibitors of plant signaling molecules may be novel controlling chemicals for *R. pseudosolanacearu*m infections.

## MATERIALS AND METHODS

### Bacterial strains and culture conditions

*The R. pseudosolanacearum* strains listed in Table 2 were OE1-1 derivatives. *E. coli* strains DH12S (Invitrogen, USA), S17-1 (42), and BL21(DE3) (43) were used for plasmid construction, conjugational transfer of plasmids, and overexpression of recombinant proteins, respectively. *E. coli* was grown in Luria–Bertani (L.B.) medium at 37°C. *R. pseudosolanacearum* was grown at 28°C in a rich B medium or hydroponic plant culture medium containing 2% sucrose (19).

**TABLE 2:** *R. pseudosolanacearum* strains used in this study

Strain	Relative characteristics	References
OE1-1	Wild-type race 1 biovar 3	(9)
RK5046	OE1-1 hrpB-lacZYA	(19)
RK5050	OE1-1 popA-lacZYA	(19)
RK5496	RK5046 ΔprhA	This study
RK5512	RK5046 Δrsc1598	This study
RK6104	RK5050 Δrsc1598	This study
RK6029	RK5050 Δrsc1075	This study
RK6088	RK5050 Δrsc0039	This study
RK6044	RK5050 Δrsp1676	This study

### Chemicals

The organic acids and sugars used in this study were of analytical grade and purchased from FUJIFILM Wako Pure Chemical Corporation.

### β-Galactosidase assay

β-Galactosidase assays were performed as described previously (44). Enzyme assays were expressed in Miller units (45). Each assay was independently repeated twice, and each trial included at least three replicates. The results are expressed as the mean ± standard deviation (SD). The data were analyzed using an Excel spreadsheet, and SPSS for Windows (SPSS, USA) was used to analyze the differences between the various treatments to assess the significance level. The distribution of the data set is shown as a boxplot. Box represents the interquartile range (IQR). The bottom and top ends of the box represent the first and third quartile, respectively. The lines inside the box represent the median values of the data. The bottom and top whiskers indicate minimum and maximum values, respectively. A value is regarded as an outlier if the data points lie between 1.5 and three times the IQR above the third quartile or below the first quartile.

### Resting cell assay

Overnight-grown *R. pseudosolanacearum* cells (0.2 mL) were inoculated into 4 mL of B media and incubated for 4–5 h at 28°C. When the culture’s OD_600_ reached 0.5 ± 0.1, the cells were harvested, washed twice with sterile 10 mM MgSO_4_, and resuspended in one-quarter strength M63 media without a carbon source (1/4 M63 buffer; 25 mM KH_2_PO_4_, 3.75 mM (NH_4_)_2_SO_4_, 0.25 mM MgSO_4_, 0.45 µM FeSO_4_) to 1.0 OD_600_. The induction reaction was performed in a 1-mL reaction mixture with 1/4 M63 buffer. The reaction continued by shaking with a Deep Well Maximizer (TAITEC, Japan) at 25°C for 20 h. Subsequently, 0.1 mL of cells was used for the reaction; 0.1 mL of tobacco extracts or 20 µL of pure chemicals was added to the reaction. After the induction reaction, 0.2 mL of mixture was transferred to a fresh tube with 0.8 mL of Z buffer (60 mM Na_2_HPO_4_, 40 mM NaH_2_PO_4_, 10 mM KCl, 1 mM MgSO_4_, and 50 mM 2-mercaptoethanol). SDS and chloroform were added at final concentrations of 0.001% and 2%, respectively, to disrupt the cells, and the β-galactosidase activity was measured as described previously.

### Viability test of *R. pseudosolanacearum* strain

Freshly prepared cells were incubated with malic acid (0.5 mM) and sucrose (0.01%) at room temperature for 20 h in a 1-mL reaction mixture. The cells were then serially diluted. Diluted cells (100 µL) were spread on the rich agar media with polymyxin B. After a 2-day incubation at 28°C, the number of colonies on the agar plates was counted. The experiments were repeated at least three times.

### Preparation of tobacco extracts

*N. benthamiana* seeds were soaked in 50% ethanol for 2 min and incubated in a sterilizing solution (10% bleach, 0.04% Tween X-100) for 5 min. After rinsing five times with sterilized distilled water, the seeds were placed on plant sucrose media with 0.8% agar (19) for germination at 26°C on a 16:8-h day:night cycle. Two-week-old tobacco seedlings (wet weight approximately 0.2 g) were placed into a 2-mL screw-capped tube containing a zirconia bead (φ5 mm) and 0.6 mL of extraction buffer (25 mM KH_2_PO_4_, 3.75 mM (NH_4_)_2_SO_4_, 0.25 mM MgSO_4_, 0.45 µM FeSO_4_). When extracts from the leaves and roots were required, the leaves and roots were separated and placed into different tubes. Plant seedlings were smashed by beads beating at 4,500 rpm for 45 s using a cell disruptor (Micro Smash MS-100, TOMY, Japan). The soluble and insoluble fractions of the crushed extracts were separated by centrifugation at 17,400 *g* for 5 min.

### Solid-phase extraction (SPE)

The Sep-Pak Plus C_18_ Cartridge (Waters, USA) was washed with methanol (4.2 mL) and equilibrated with the extraction buffer (4.2 mL). Soluble tobacco extracts were applied to the cartridge and washed with 1.4 mL of extraction buffer. The bound components were eluted using a stepwise gradient of methanol (50% and 100%). The flow-through, wash, and two-elution fractions were concentrated and resuspended in 50 µL of DMSO.

Sep-Pak Plus CM and QMA cartridges were connected in tandem and equilibrated with 4.2 mL of balance buffer (10 mM ammonium acetate, pH 5.5). After the samples were loaded onto these two cartridges, the cartridges were washed with 2 mL of wash buffer (10 mM ammonium acetate, pH 5.5 in 20% acetonitrile) and disconnected into individual cartridges. The two cartridges were slowly eluted with 6 mL (six times, 1 mL each time) of eluent buffer (10 mM ammonium acetate, pH 5.5, and 1 M NaCl in 20% acetonitrile).

### Separation of organic acids

Organic acids were analyzed by strong ion-exchange chromatography using a post-column method with a pH indicator. Organic acids were separated with a strong (sulfonic acid) cation exchanger TSKgel SCX (30 cm ×7.8 mm, 5-µm particle size, TOSOH, Japan) connected to a guard column using 3 mM HClO_4_ as the eluent at a flow rate of 1 mL/min. The reaction reagent, 0.2 mM bromothymol blue (BTB) in 15 mM Na_2_HPO_4_, flowed at 1.5 mL/min using another pump. The reaction reagent solution was basic to dissociate the pH indicator BTB (I^−^ and H^+^). The reaction reagent solution became acidic when it was mixed with the eluent in the mixing module. The mixing causes the pH indicator to undergo the reaction I^−^ + H^+^ → IH and changes its color. Color change was detected using a visible detector (445 nm). Pure organic acids were separated using a post-column method. We identified the organic acids in the tobacco extracts based on the retention times of the pure standards.

### Separation of sugars

The sugars were separated on a Sugar-D column (4.6 mm × 250 mm, particle size, Nacalai Tesque, Japan) using 75% acetonitrile as the mobile phase at a flow rate of 1 mL/min and detected using a refractive index detector.

### Purification of Rsc1598 periplasmic domain

A 0.65-kb DNA fragment containing the periplasmic domain (amino acid residues 99–319) of Rsc1598 was amplified from the chromosomal DNA of *R. pseudosolanacearum* OE1-1 using a pair of primers, rsc1598A102; 5′-GGAGATATACATATGCATGCTACCGAGGTGCAGCA-3′ and rsc1598B102; 5′-GTGGTGGTGGTGGTGGGGCAGGTTGGACAGCGCCG-3′. The amplified fragment was cloned into pET21a(+) using an infusion system to construct ppet1598. *E. coli* BL21(DE3) cells transformed with ppet1598 were incubated in 250 mL of L.B. media at 37°C until the cell density at 600 nm reached 0.6. IPTG (final concentration, 0.1 mM) was added, and the cells were incubated overnight at 28°C. Cells were harvested, resuspended in 20 mL of lysis buffer (50 mM NaH_2_PO_4_, pH 8.0, 300 mM NaCl, and 10 mM imidazole), and sonicated. After removing the cell debris, the supernatant was mixed with 1 mL of Ni-NTA agarose slurry (Qiagen, Germany) at 4°C for 1 h. Proteins bound to Ni-NTA were eluted using elution buffer (50 mM NaH_2_PO_4_, pH 8.0, 300 mM NaCl, and 250 mM imidazole).

### Isothermal titration calorimetry (ITC)

The purified proteins were dialyzed against 50 mM sodium phosphate buffer (pH 7.6) and diluted to 40 µM. ITC data were obtained using a MicroCal ITC200 instrument (Malvern Panalytical, Malvern, UK). The experiments were performed at 25°C using different concentrations of organic acids. The first injection was 0.4 µL and then 18 injections of 2.0 µL with a delay of 120 s between injections. The syringe paddle was continuously rotated at 750 rpm in the sample pool. ITC-binding isotherms were analyzed using MicroCal PEAQ-ITC Analysis Software Version 1.22 (Malvern Panalytical, UK). The original data were integrated, and the baseline and normalized active-component concentrations were adjusted. The data were analyzed using a set of site-binding models.

## SUPPLEMENTAL MATERIAL

Figure S1 jb.00273-24-s0001.pdf Model of hrp gene regulatory cascade in *R. pseudosolanacearum*.

Figure S2 jb.00273-24-s0002.pdf Cell viability after 20-h incubation.

Figure S3 jb.00273-24-s0003.pdf Preparation and separation of tobacco extracts.

Figure S4 jb.00273-24-s0004.pdf Induction of hrpB in growing cells.

Figure S5 jb.00273-24-s0005.pdf Isothermal titration calorimetry analysis of ligands binding to the periplasmic domain of Rsc1598.

ASM does not own the copyrights to Supplemental Material that may be linked to, or accessed through, an article. The authors have granted ASM a non-exclusive, world-wide license to publish the Supplemental Material files. Please contact the corresponding author directly for reuse.
