# Supplementary material for: Sucrose and malic acid in the tobacco plant induce hrp regulon in a phytopathogen Ralstonia pseudosolanacearum
Source: J Bacteriol. 2025 Feb 4;207(3):e00273-24. doi: 10.1128/jb.00273-24 (PMC11925246; doi:10.1128/jb.00273-24)
Supplement: Figure S1 — Model of hrp gene regulatory cascade in R. pseudosolanacearum. [file jb.00273-24-s0001.pdf]

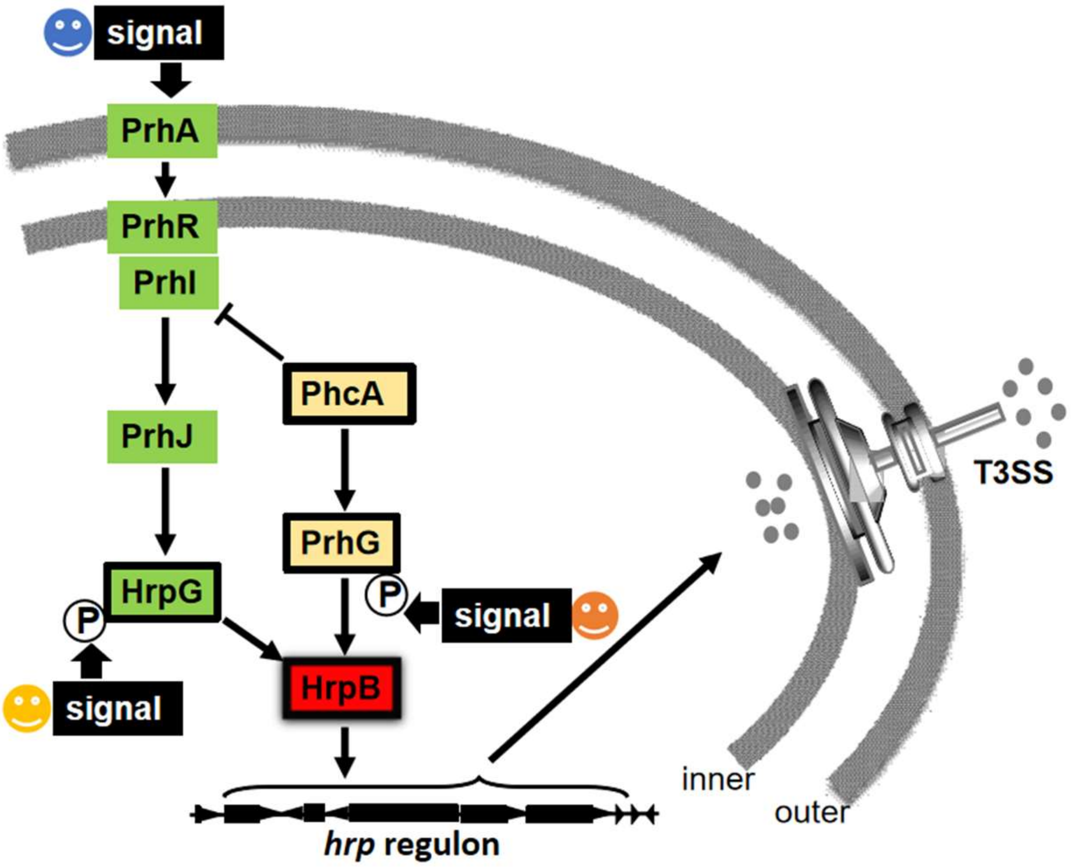

FIG. S1. Model of *hrp* gene regulatory cascade in *R. pseudosolanacearum* induced in response to potential plant signaling molecules. Blue, yellow and orange smiley faces represent potential plant signaling molecules. The P flag represents phosphate transfer.
