# Supplementary material for: Sucrose and malic acid in the tobacco plant induce hrp regulon in a phytopathogen Ralstonia pseudosolanacearum
Source: J Bacteriol. 2025 Feb 4;207(3):e00273-24. doi: 10.1128/jb.00273-24 (PMC11925246; doi:10.1128/jb.00273-24)
Supplement: Figure S2 — Cell viability after 20-h incubation. [file jb.00273-24-s0002.pdf]

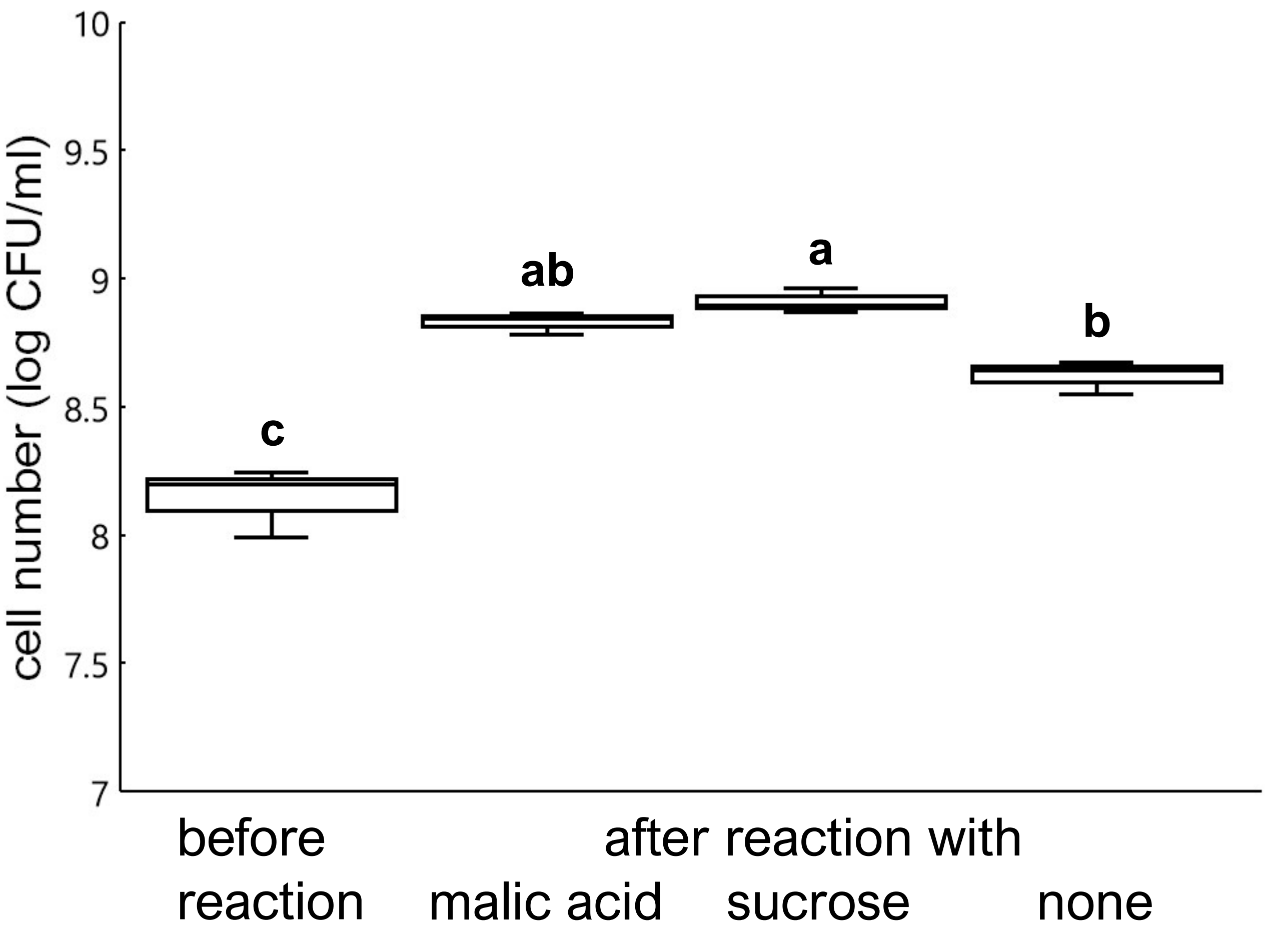

FIG. S2. Cell viability after 20-h incubation. Freshly prepared resting cells of RK5512 (*hrpB-lacZ* Δ*rsc1598*) were incubated with malic acid (0.5 mM) and sucrose (0.01%) at room temperature for 20 h in a 1-ml reaction mixture. The cells were serially diluted. Diluted cells (100 µl) were spread on the rich agar media with polymyxin B. After a two-day incubation at 28 °C, the number of colonies on the agar plates was counted. Box plots show the medians (horizontal line in the box), 25 and 75% quartiles, and max/min values. The experiments were repeated at least three times. Letters indicate a significant difference between treatments ($P<0.05$ Tukey HSD test).
