# Supplementary figures and images for: Sucrose and malic acid in the tobacco plant induce hrp regulon in a phytopathogen Ralstonia pseudosolanacearum
Source: J Bacteriol. 2025 Feb 4;207(3):e00273-24. doi: 10.1128/jb.00273-24 (PMC11925246; doi:10.1128/jb.00273-24)

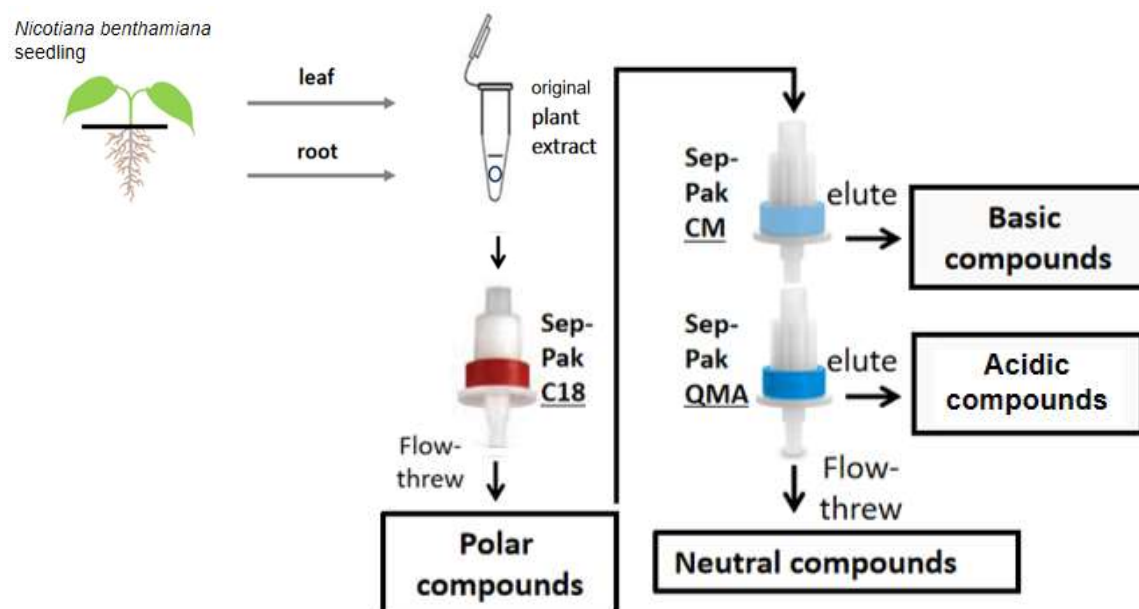

FIG. S3. Preparation and separation of tobacco extracts.

Supplement: Figure S3 — Preparation and separation of tobacco extracts. [file jb.00273-24-s0003.pdf]
