# Supplementary material for: Sucrose and malic acid in the tobacco plant induce hrp regulon in a phytopathogen Ralstonia pseudosolanacearum
Source: J Bacteriol. 2025 Feb 4;207(3):e00273-24. doi: 10.1128/jb.00273-24 (PMC11925246; doi:10.1128/jb.00273-24)
Supplement: Figure S4 — Induction of hrpB in growing cells. [file jb.00273-24-s0004.pdf]

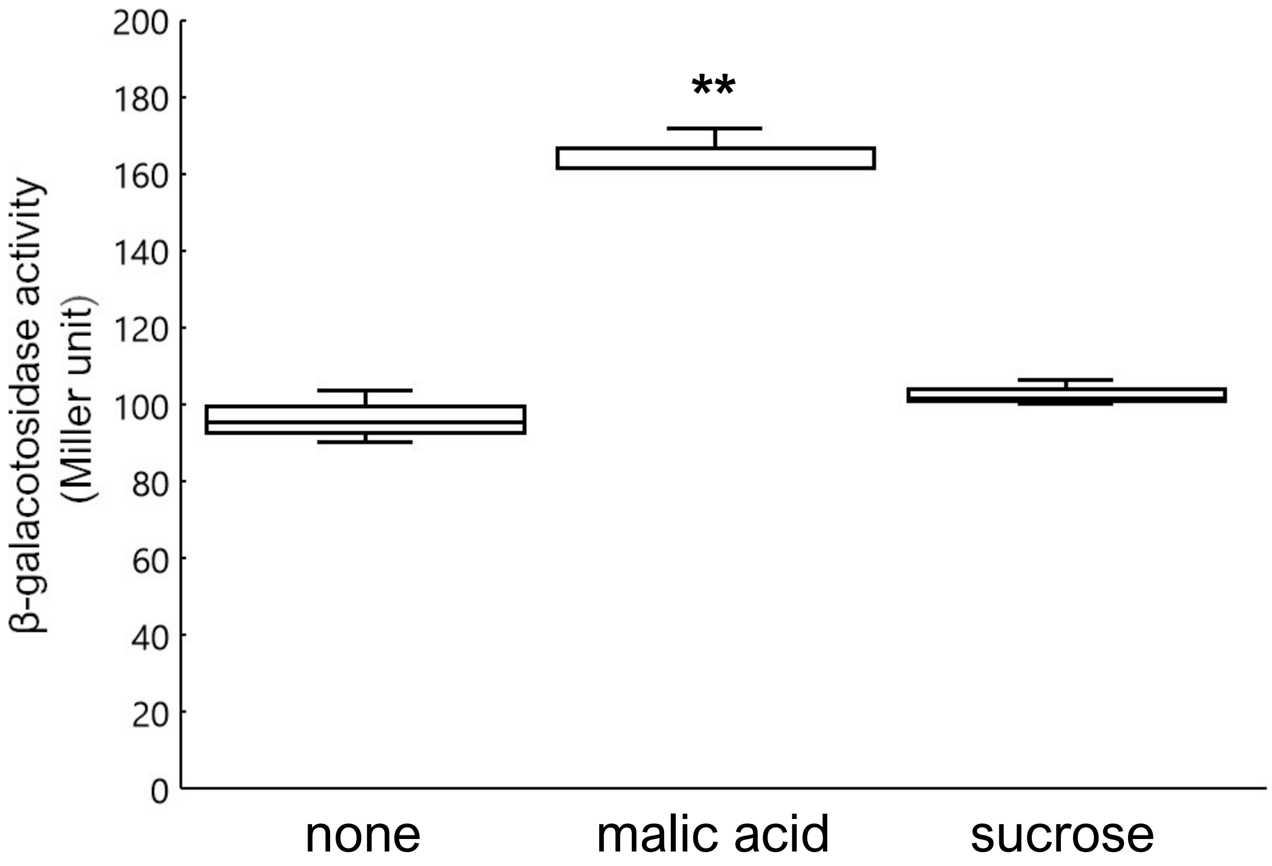

FIG. S4. Induction of hrpB in growing cells. Overnight grown RK5046 (*hrpB-lacZ*) cells (20 µl) were inoculated into 2-ml fresh quarter strength M63 minimal medium with 0.25 % glucose as a carbon source. Malic acid (0.5 mM) or sucrose (0.01%) was added to induce the *hrpB-lacZ* expression. After incubation at 28 °C for 5 h, the cells were recovered, and β-galactosidase activity was measured. Box plots show the medians (horizontal line in the box), 25 and 75% quartiles and max/min values. The experiments were repeated at least three times. ** indicates a statistically significant difference from none at $p<0.01$.
