# Supplementary material for: Sucrose and malic acid in the tobacco plant induce hrp regulon in a phytopathogen Ralstonia pseudosolanacearum
Source: J Bacteriol. 2025 Feb 4;207(3):e00273-24. doi: 10.1128/jb.00273-24 (PMC11925246; doi:10.1128/jb.00273-24)
Supplement: Figure S5 — Isothermal titration calorimetry analysis of ligands binding to the periplasmic domain of Rsc1598. [file jb.00273-24-s0005.pdf]

(A)

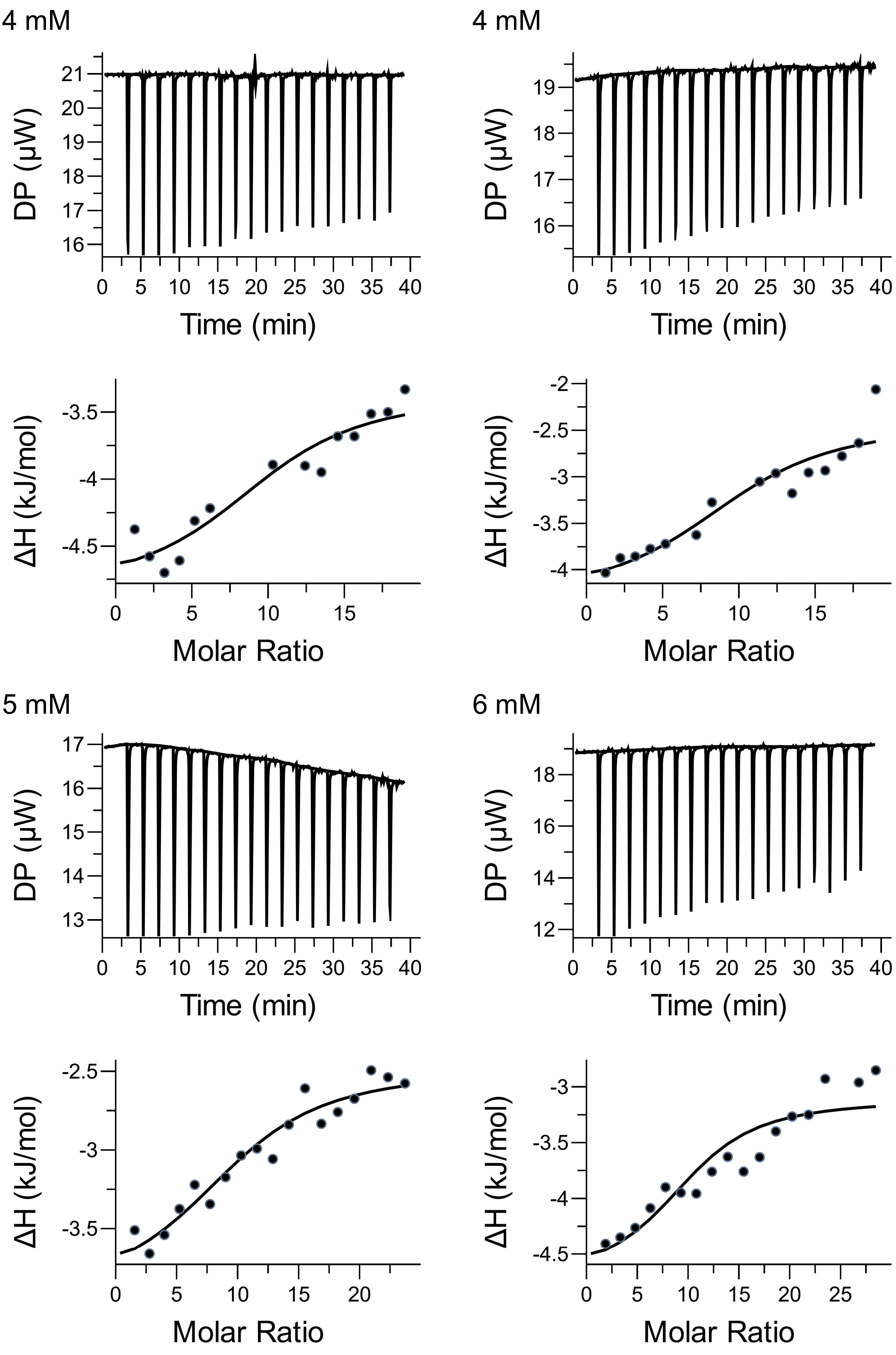

DL-malic acid

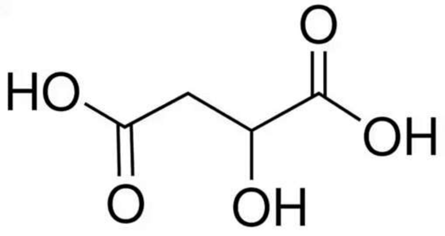

(B)

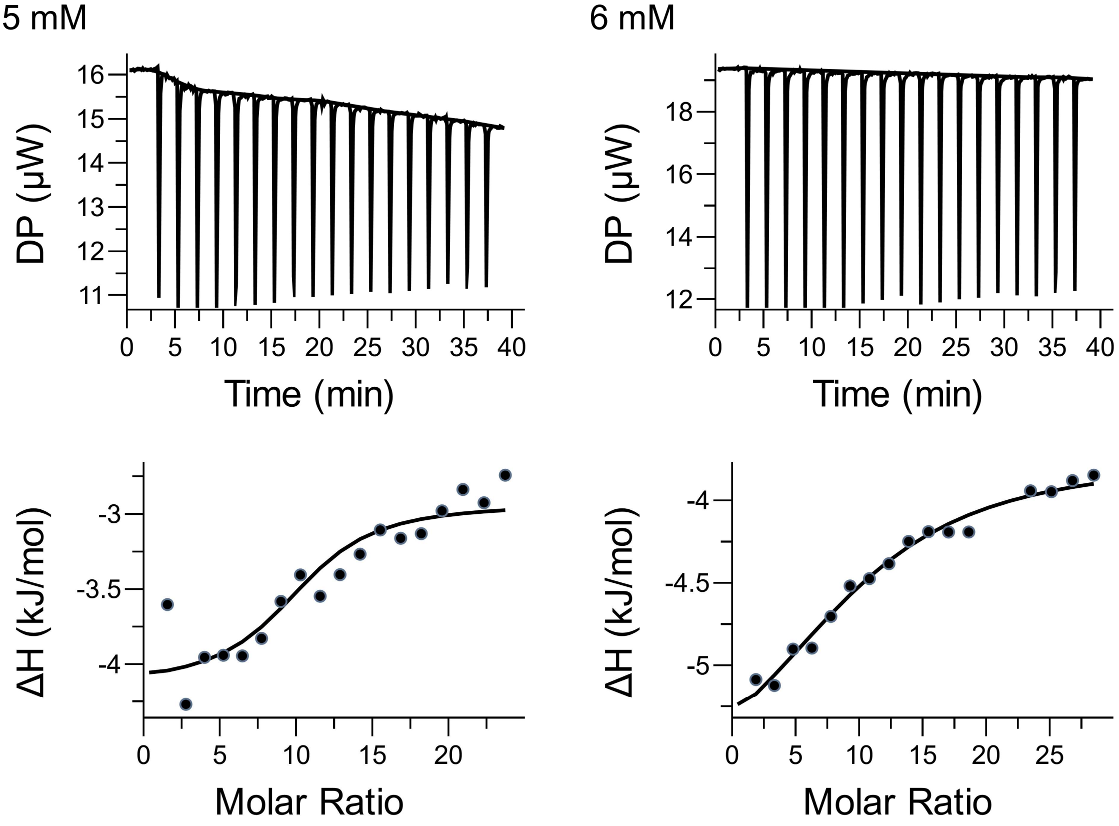

succinic acid

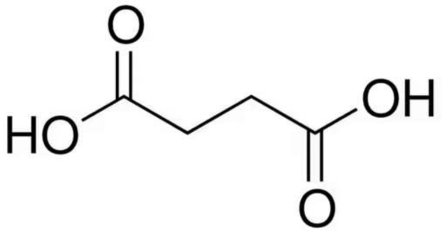

(C)

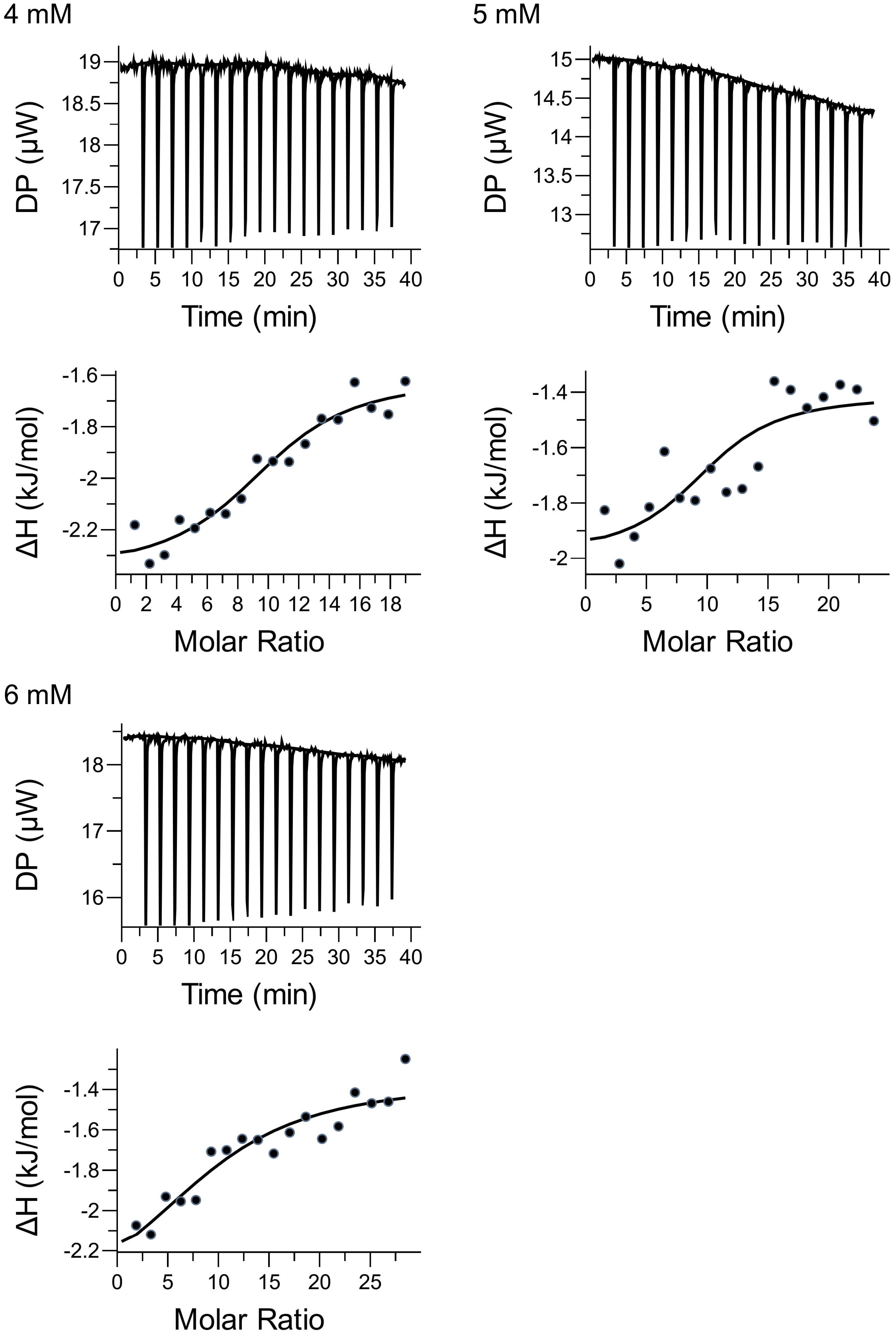

L-pyroglutamic acid

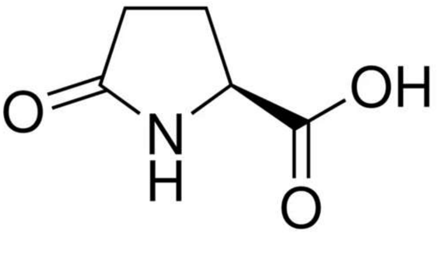

(D)

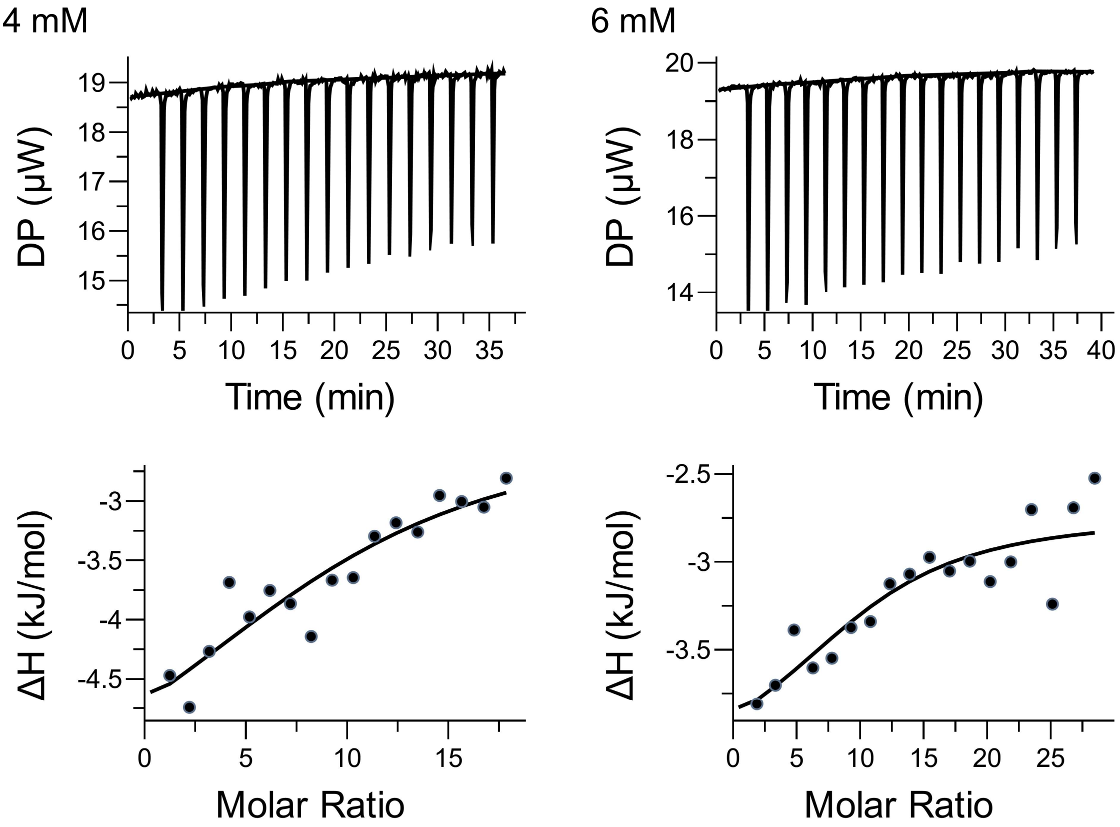

oxalic acid 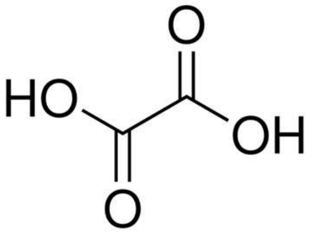

(E)

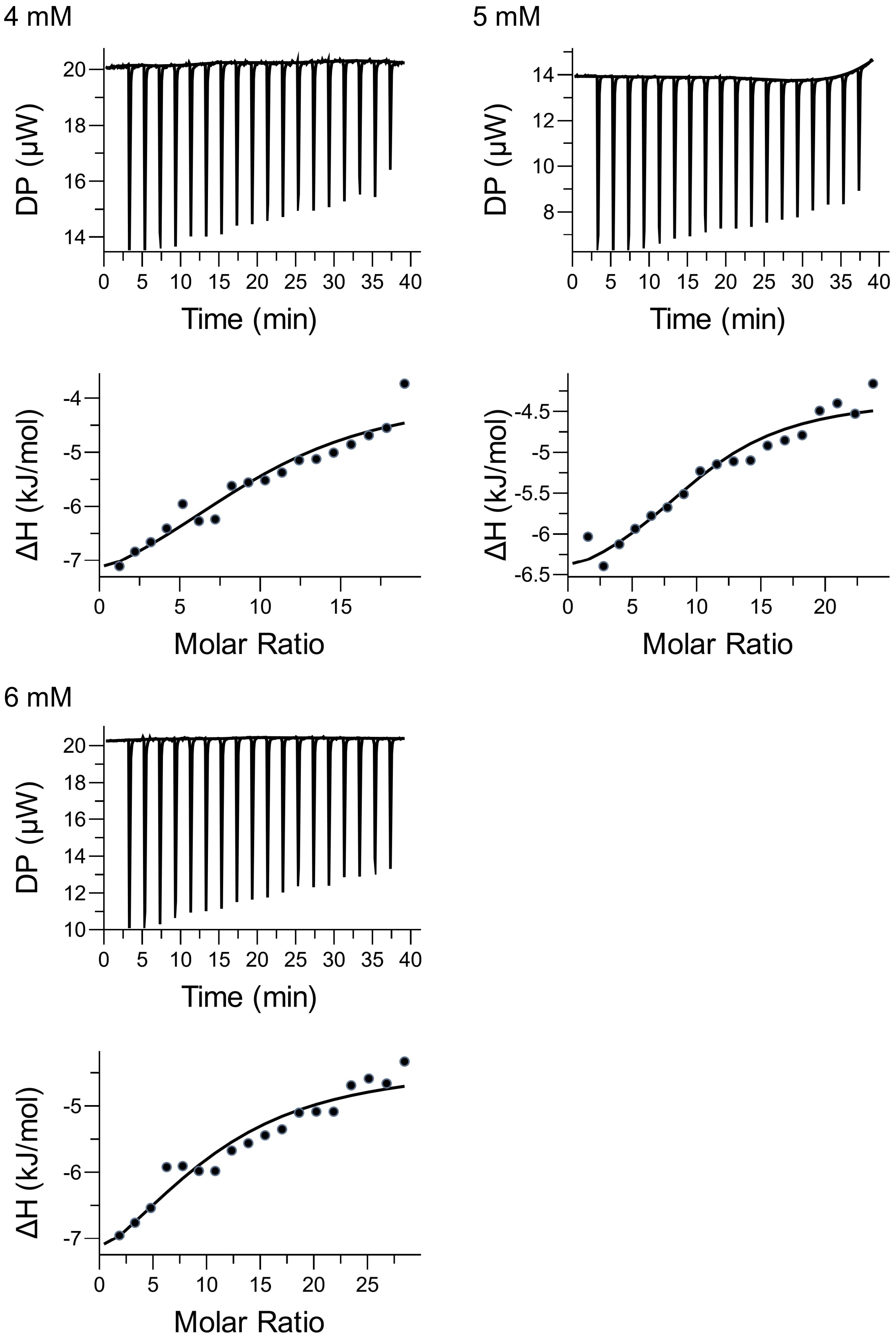

citric acid

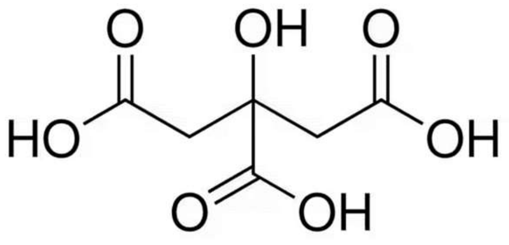

(F)

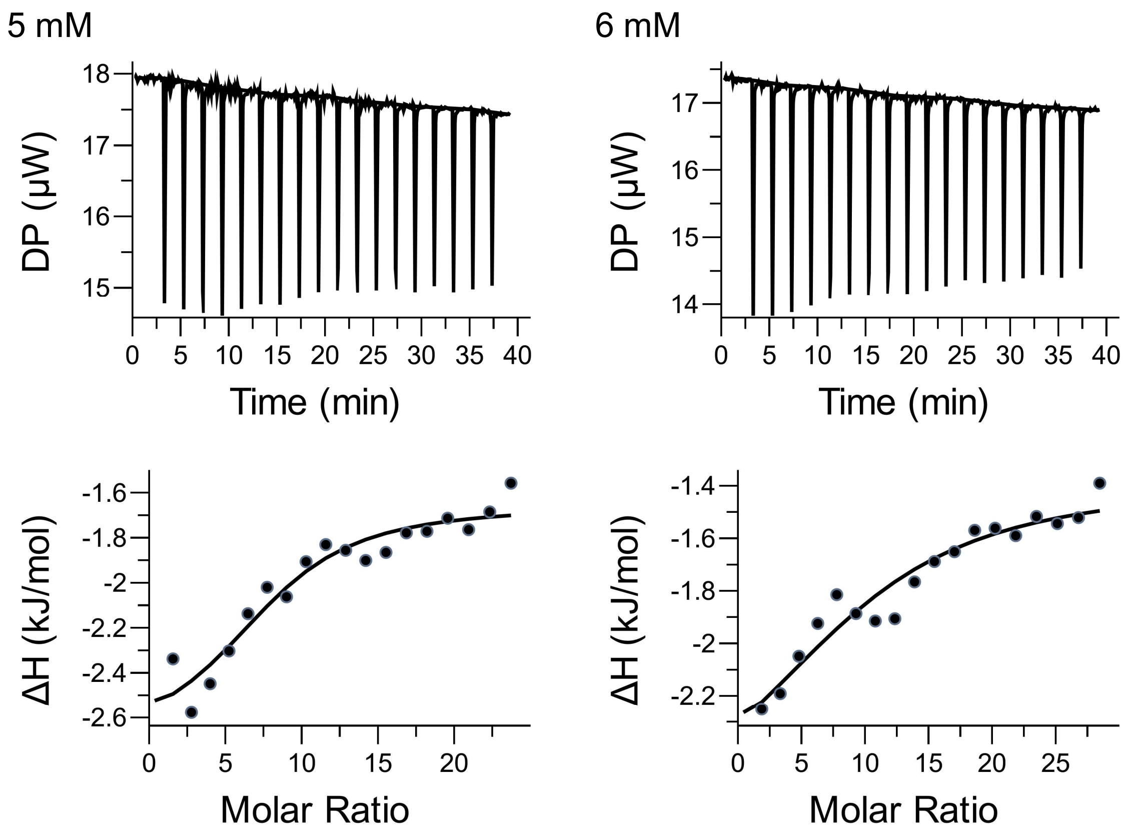

formic acid

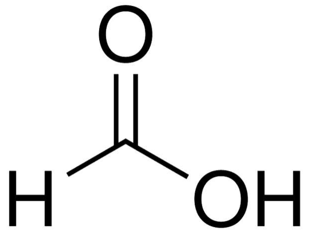

(G)

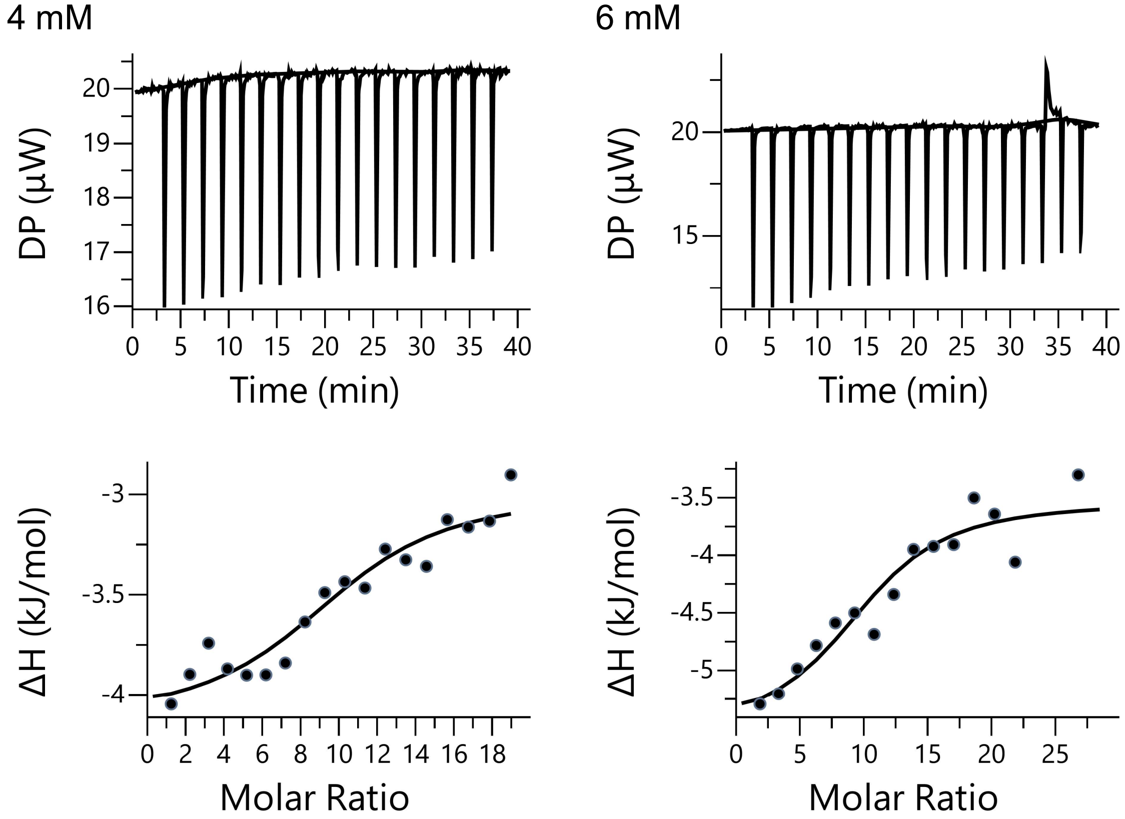

fumaric acid

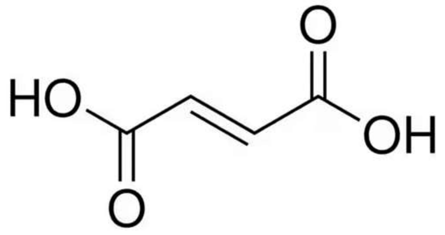

FIG. S5. Isothermal titration calorimetry analysis of ligands binding to the periplasmic domain of Rsc1598. Raw titration data obtained using a MicroCal ITC200 indicated: (A) DL-malic acid, (B) succinic acid, (C) L-pyroglutamic acid, (D) oxalic acid, (E) citric acid, and (F) formic acid. Purified periplasmic domain (40 μM) of Rsc1598 was used. The top panel shows the lattice data baseline-corrected and mapped using Origin software. The bottom panel shows the integrated and dilution-corrected peak areas for the raw data. Data were fitted with the “one binding site model” of the MicroCal PEAQ-ITC Analysis Software Version 1.22 (Malvern Panalytical, UK). The ligand concentration is indicated at the top. The structure of the ligand is also shown.
